# A Scoping Review Investigating the “Gene-Dosage Theory” of Mitochondrial DNA in the Healthy Skeletal Muscle

**DOI:** 10.3390/ijms24098154

**Published:** 2023-05-02

**Authors:** Zandra Overgaard Pedersen, Britt Staevnsbo Pedersen, Steen Larsen, Tina Dysgaard

**Affiliations:** 1 Copenhagen Neuromuscular Center, Department of Neurology, Copenhagen University Hospital, Rigshospitalet, 2100 Copenhagen, Denmark; 2 Steno Diabetes Center Copenhagen, 2730 Herlev, Denmark; 3 Xlab, Center for Healthy Aging, Department of Biomedical Sciences, Faculty of Health and Medical Sciences, University of Copenhagen, 2100 Copenhagen, Denmark; 4 Clinical Research Centre, Medical University of Bialystok, 15-089 Bialystok, Poland

**Keywords:** mitochondrial DNA, mtDNA, aerobic exercise, mitochondrial content, mitochondrial adaptions, citrate synthase, CS, electron transport chain

## Abstract

This review provides an overview of the evidence regarding mtDNA and valid biomarkers for assessing mitochondrial adaptions. Mitochondria are small organelles that exist in almost all cells throughout the human body. As the only organelle, mitochondria contain their own DNA, mitochondrial DNA (mtDNA). mtDNA-encoded polypeptides are subunits of the enzyme complexes in the electron transport chain (ETC) that are responsible for production of ATP to the cells. mtDNA is frequently used as a biomarker for mitochondrial content, since changes in mitochondrial volume are thought to induce similar changes in mtDNA. However, some exercise studies have challenged this “gene-dosage theory”, and have indicated that changes in mitochondrial content can adapt without changes in mtDNA. Thus, the aim of this scoping review was to summarize the studies that used mtDNA as a biomarker for mitochondrial adaptions and address the question as to whether changes in mitochondrial content, induce changes in mtDNA in response to aerobic exercise in the healthy skeletal muscle. The literature was searched in PubMed and Embase. Eligibility criteria included: interventional study design, aerobic exercise, mtDNA measurements reported pre- and postintervention for the healthy skeletal muscle and English language. Overall, 1585 studies were identified. Nine studies were included for analysis. Eight out of the nine studies showed proof of increased oxidative capacity, six found improvements in mitochondrial volume, content and/or improved mitochondrial enzyme activity and seven studies did not find evidence of change in mtDNA copy number. In conclusion, the findings imply that mitochondrial adaptions, as a response to aerobic exercise, can occur without a change in mtDNA copy number.

## 1. Introduction

Mitochondria are double membrane organelles that are responsible for the cellular energy production of adenosine triphosphate (ATP) by oxidative phosphorylation (OXPHOS) [1]. In addition, mitochondria play a central role in numerous cellular functions important for regulating cell homeostasis and survival [2]. Thus, the dysfunction of mitochondria results in impaired cellular energy production and affects multiple regulatory factors central to cell survival, which can result in neurodegenerative disorders [3,4], cancer [5,6] and development of metabolic disorders [7,8]. Due to mitochondria’s regulating role in essential cellular regulatory mechanisms, the interest in the regulation of mitochondria has been increasing since the discovery of mitochondrial adaptions to exercise in 1967 [9]. Mitochondria exist in almost all cells throughout the human body. The number of mitochondria can be up to 10-fold higher per cell in highly oxidative tissues, such as skeletal and cardiac muscle, compared with cells with lower oxidative demand, such as kidney and skin cells. Mitochondria adapt in response to changes in energy demand, where aerobic exercise is one of the most potent ways to induce mitochondrial adaptions in the skeletal muscle [10]. Some of the main adaptions in response to aerobic exercise are improvements in mitochondrial function, biogenesis and content [10]. Since these parameters can be studied directly via skeletal muscle biopsies that can be taken serially, the response to aerobic exercise has been an important intervention when studying mitochondrial adaptions in response to changes in energy demand [11].

The widely accepted gold standard for measuring mitochondrial content is full-scale transmission electron microscopy (TEM) [12]. However, the method is not widely used since it is time-consuming, costly and not widely accessible [12]. Due to the complexity of TEM, different approaches can be used to assess mitochondrial volume. One approach to assess mitochondrial volume has been by measuring the content of mitochondrial membrane proteins. Porin is a protein present in the outer membrane of the mitochondria, whereas cardiolipin is a phospholipid located in the inner membrane of the mitochondria and both have been used as a quantitative marker for the amount of mitochondrial membrane and thus mitochondrial volume [13,14].

Another approach to assess mitochondrial adaptions has been by measuring mitochondrial enzyme activity. Mitochondrial enzyme activity is expressed through the mitochondrial electron transport chain (ETC). ETC is located in the mitochondrial inner membrane and consists of four enzyme complexes that transfer electrons from electron donors, for instance NADH and FADH2, to electron acceptors, such as oxygen [15,16]. ETC activity influences a diversity of processes beyond energy balance, such as reactive oxygen species (ROS) production [16,17]. Enzymes in the ETC are often used as biomarkers for mitochondrial adaptions and as indicators of mitochondrial oxidative capacity [11,18]. Determination of the complex activities in ETC can be assessed by spectrophotometric assays. Specific enzymes can be used as an expression for complex activities, such as ubiquinol oxidase, cytochrome c oxidase and NADH oxidase as indicators of complex I activity and succinate dehydrogenase as indicators of complex II activity [19,20]. Another widely used enzyme that is used to address mitochondrial adaptions is citrate synthase [21,22]. Citrate synthase is an enzyme exclusively located in the mitochondria. Citrate synthase is the first of eight enzymes in the tricarboxylic acid cycle (TCA) and catalyses the first step in TCA, which is an essential metabolic pathway in ATP production [23]. The activity of citrate synthase has been shown to adapt to changes in energy demand and thus the demand on mitochondrial content. Therefore, the activity of citrate synthase is often used as a biomarker for mitochondrial content [24,25].

It is well documented that aerobic exercise leads to changes in mitochondrial volume and function and induce adaptions in mitochondrial biogenesis [9,21,26,27,28,29,30]. Mitochondrial biogenesis is defined as an increase in the mitochondrial mass or mitochondrial division of the pre-existing mitochondria; however, there is no widely accepted definition of which biological biomarkers that should be used as a marker for mitochondrial biogenesis [31,32]. Proteins that are used as indicators of mitochondrial biogenesis include, peroxisome proliferator-activated receptor gamma coactivator 1-alpha (PGC-1*a*) [33,34], peroxisome proliferator-activated receptor gamma (PPAR*y*) [35,36], P53 [37] and nuclear respiratory factor (NRF-1) [38]. PGC-1*a* in the skeletal muscle is activated and increased after exercise [39]. PGC-1*a* activates PPAR*y* and NRF-1 and mediates regulations of several mitochondrial pathways, which may have an impact on mitochondrial content and function, and could therefore be used as a biomarker of mitochondrial content as a response to aerobic exercise [39,40].

Studies have implied that mitochondrial adaptions occur simultaneously with adaptions in the number of mtDNA [41,42,43,44]. Therefore, mtDNA has been thought to change proportionally with mitochondrial biogenesis, function and content and has been used as a biomarker for mitochondrial content in both the healthy, as well as the diabetic skeletal muscle [45,46,47,48]. However, some studies have not been able to find this association. In fact, studies have found that biomarkers such as citrate synthase, ETC and cardiolipin can change while mtDNA content remains unchanged [49,50,51]. These findings have questioned the theory of a direct 1:1 relationship between mitochondrial content and mtDNA, often pronounced as “the gene-dosage theory”, where mtDNA replication is a necessary mechanism for exercise-induced mitochondrial adaptions.

Therefore, this scoping review aimed to summarize the studies that used mtDNA as a biomarker for mitochondrial adaptions and address to what extent mtDNA resembles mitochondrial content in response to aerobic exercise in healthy skeletal muscle.

## 2. Methods

This scoping review was conducted according to the PRISMA extension for scoping reviews (PRISMA-ScR) [52].

### 2.1. Search Strategy

The following two databases were searched for literature: PubMed and Embase. A pre-specified literature search was conducted in December 2022 without any search limitations. A librarian checked the search strategy. Reference lists were reviewed for additional literature. The search strategy is presented in Appendix A.

### 2.2. Eligibility Criteria

Interventional studies were included if they reported an intervention of vigorous aerobic exercise and if healthy adults were included. The aerobic exercise protocol and intensities should be clearly described. Levels of mtDNA should be measured by muscle biopsies in the healthy skeletal muscle pre- and postintervention. Only studies in English language were included.

### 2.3. Exclusion Criteria

(i) Animal studies, (ii) acute bout exercises and (iii) studies not presenting data for mtDNA measures by values or figures.

### 2.4. Study Selection

The search results were downloaded and imported to reference manager Zotero. Duplicates were checked and removed manually by one author (ZOP). One author (ZOP) independently screened and selected the papers on title and abstract. Two authors (ZOP, BP) reviewed full-text articles and selected them according to eligibility criteria. Disagreements were solved by discussion; if an agreement could not be reached a third reviewer was consulted (TD).

### 2.5. Data Extraction

All data were extracted into a special designed Excel spreadsheet to manage the data. One author extracted the data (ZOP). Two authors (TD, BP) checked the data extraction to ensure accuracy. The following data were extracted: 

*Study characteristics:* Authors, publication year, study design, number of participants, sex, age, training status, body mass index (BMI) or kilogram (Kg). 

*Descriptions of exercise program:* Exercise mode, sessions per week, training duration, minutes per session and work-bout intensity. mtDNA levels and relevant biomarkers for mitochondrial adaptions, insulin resistance and oxygen uptake pre- and postintervention were extracted.

### 2.6. Outcome Measures

The outcomes of interest were mtDNA in response to aerobic exercise, mtDNA levels in comparison with other biomarkers for mitochondrial content, oxygen uptake and insulin resistance pre- and postintervention.

### 2.7. Quality Appraisal and Interrater Reliability

The PEDro scale is an 11-point scale designed to rate the quality of clinical trials indexed in the Physiotherapy Evidence Database [53]. The score ranges from 0–11, with highest score representing the highest methodological quality. The PEDro scale is created from the Delphi list and comprises items regarding internal and external validity [53]. Interrater reliability was calculated using the Cohens kappa coefficient [39] in selecting full-text articles between the two authors (ZOP, BP).

## 3. Results

### 3.1. Study Selection

**Figure 1:**
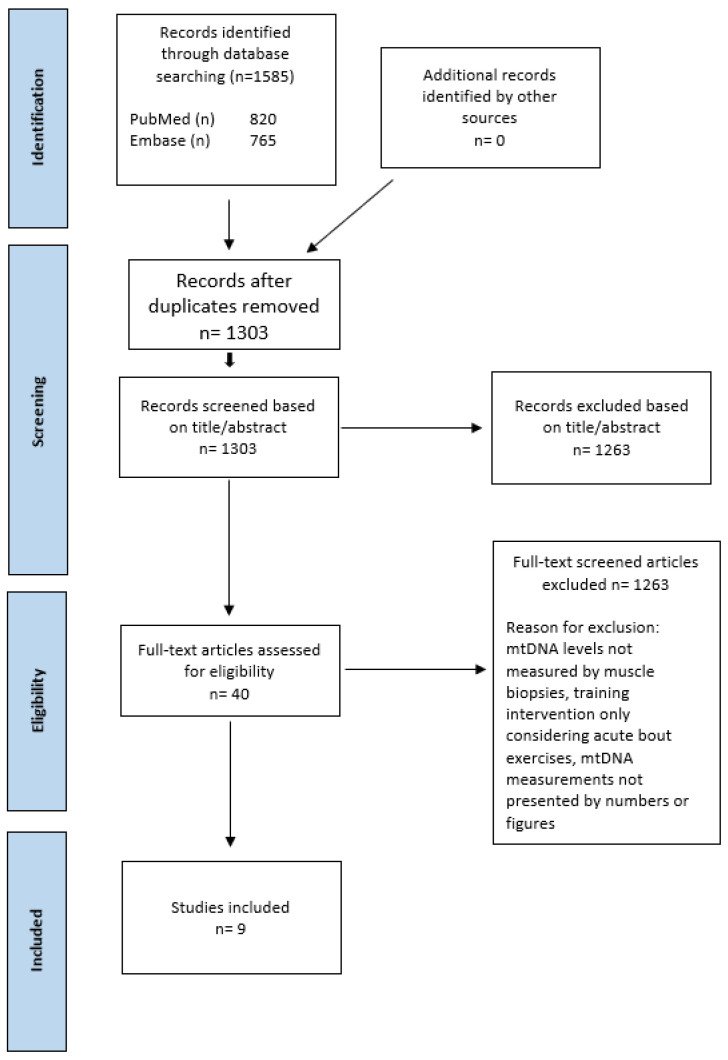
PRIMA flowchart.

The search strategy yielded a total of 1585 results. After duplicates were removed, 1303 records were screened on title and abstract. A total of 40 articles were assessed and full-text screened for eligibility. Nine studies were included for detailed analysis [45,48,54,55,56,57,58,59,60]. A PRISMA flowchart [61] of the study selection is illustrated in Figure 1. Two publications used data from the same dataset [45,55]. Menshikova et al. [45] compiled their data separately for males and females. Data will therefore be presented independently. One study investigated the effect of environmental acclimation and compared a 3-week training period in 7° vs. 20° [58]. Data for the included subjects will be presented combined. Interrater reliability regarding full-text screening had Cohen’s kappa value of 0.7, indicating moderate agreement between the authors.

### 3.2. Study Characteristics (Table 1 and Table 2)

The nine included studies comprehended a total of 155 sedentary or untrained subjects, with a mean age of 38.4 years. Overweight, as defined as BMI ≥ 25 [62], was present in 124 subjects. mtDNA measurements by muscle biopsies were measured in 126 subjects. Work-bout duration of the training sessions ranged from 30–60 min per session, at an exercise intensity from moderate to high. The training interventions consisted of cycling, walking, treadmill and knee extensor training. The total number of training sessions ranged from 20–80. Specifications for training interventions are illustrated in Table 2.

**Table 1:** Study characteristics.

Study	Study Design	n	Age	Sex	Training Status	BMI/kg Baseline
Menshikova et al., 2005 [45] Males	NCT	7/5 *	39.4 **	M	Sedentary	31.7 **
Menshikova et al., 2005 [45] Females	NCT	7/6 *	38.8 **	F	Sedentary	35.1 **
Menshikova et al., 2006 [54]	NCT	8	67.3	5M/3F	Sedentary	28
Menshikova et al., 2007 [55]	CT	7	40.6	4M/3F	Sedentary	34.1
Majerczak et al., 2012 [60]	NCT	10	22.9	M	Untrained	23.6
Egan et al., 2013 [59]	NCT	8	23	M	Sedentary	23.6
Shute et al., 2020 [58]	RCT	24	27	M	Untrained	86.1 kg
Fritzen et al., 2019 [57]	NCT	13	22.9	M	Sedentary	22
Murakami et al., 2002 [56]	NCT	55/29 *	20.5 **	M	Sedentary	62.4 kg **
Toledo et al., 2008 [51]	RCT	16/9 ***	42.4	3M/6F	Sedentary	34.8

All data are represented as means. Variability measures are excluded from the table due to substantial dissimilarity in variability measures. Abbreviations: NCT = Non-controlled trial, CT = Controlled trial, F = Females, M = Males, Kg = kilogram. * In third column, n/n denotes the number of included subjects vs. subjects where mtDNA was assessed. ** Data representing all included subjects. *** Data will be presented combined. Exercise intervention only performed by 9 subjects. This review will only manage data for these 9 subjects.

**Table 2:** Training program description.

Study	Exercise Mode	Sessions per Week	Training Duration (Weeks)	Minutes per Session	Work-Bout Intensity
Menshikova et al., 2005 [45] Males	Cycling, Treadmill or Walking	4–6	16	30–40	60–70% of maximal heart rate. Intensity increased to 75% of maximal heart rate for the last 4 weeks
Menshikova et al., 2005 [45] Females	Cycling, Treadmill or Walking	4–6	16	30–40	60–70% of maximal heart rate. Intensity increased to 75% of maximal heart rate for the last 4 weeks
Menshikova et al., 2006 [54]	Cycling, Treadmill or Walking	4–6	12	30–40	50–60% VO_2max_. Intensity increased to ~70% VO_2max_ for the last 4 weeks
Menshikova et al., 2007 [55]	Cycling, Treadmill or Walking	4–6	16	30–40	60–70% of maximal heart rate. Intensity increased to 75% of maximal heart rate for the last 4 weeks
Majerczak et al., 2012 [60]	Cycling	2	5	40	90% V_O2_ at lactate threshold
Egan et al., 2013 [59]	Cycling	7	2	60	~80% VO_2peak_
Shute et al., 2020 [58]	Cycling	5	3	60	Perceived intensity of 15 on RPE scale
Fritzen et al., 2019 [57]	Knee Extensor (Continuous and Intermittent)	4	6	40	Continuous: 70% VO_2peak_ Intermittent: 5 min 95% VO_2peak_ followed by 3 min with minimal resistance
Murakami et al., 2002 [56]	Cycling	3.5	8	60	70% of VO_2Max_
Toledo et al., 2008 [51]	Treadmill or Walking	3–5	16–20 weeks	30–40	60–70% of maximal heart rate

Abbreviations: RPE-Scale = Rated perceived exertion, VO_2max_ = Maximal oxygen consumption, VO_2peak_ = Volume of oxygen uptake during peak exercise.

### 3.3. Study Quality (Table 3)

The overall study quality scored by the PEDro scale ranged from 3 to 7 on the 11-point scale. The low scores can partly be explained by the non-controlled trial design, where randomization and concealed allocation were not possible. None of the included studies used blinding of the therapist or subjects, which is a common limitation in training studies.

The quality assessment of the studies is presented in Table 3.

**Table 3:** Quality assessment according to PEDro scale.

Study	Eligibility Criteria Specified	Randomly Allocated to Groups	Allocation Concealed	Similar Groups at Baseline	Blinding of Subjects	Blinding of Therapists	Blinding of Assessors	Measures of Key Outcome Obtained ≥ 85%	Subjects Where Outcome Measures Were Available Received Treatment or Control	Between Groups Comparisons	Point Measure and Variability
Menshikova et al., 2005 [45]	1	0	0	0	0	0	0	0	1	0	1
Menshikova et al., 2006 [54]	1	0	0	0	0	0	0	1	1	0	1
Menshikova et al., 2007 [55]	1	0	0	0	0	0	0	1	1	1	1
Majerczak et al., 2012 [60]	0	0	0	0	0	0	0	1	1	0	1
Egan et al., 2013 [59]	0	0	0	0	0	0	0	1	1	0	1
Shute et al., 2020 [58]	0	1	0	1	0	0	0	1	1	1	1
Fritzen et al., 2019 [57]	0	0	0	0	0	0	0	1	1	1	1
Murakami et al., 2002 [56]	0	0	0	0	0	0	0	0	1	1	1
Toledo et al., 2008 [51]	1	1	0	1	0	0	0	1	1	1	1

1 = Yes, 0 = No or not mentioned.

## 4. Outcomes

### mtDNA (Table 4)

mtDNA levels changed in response to exercise in two of the nine studies [54,56]. In one study, mtDNA increased by 53% [54], whereas mtDNA increased by 16% in another study [56]. In the remaining studies, mtDNA remained unchanged in the skeletal muscle in response to aerobic exercise [45,51,55,57,58,59,60].

**Table 4:** mtDNA and biomarkers of mitochondrial content and function.

Study	Biomarkers	Preintervention	Postintervention	Change
Menshikova et al., 2005 [45] Males	mtDNA, Rc Succinate oxidase, U/mU CK ETC/mtDNA ratio	1754 0.166 0.118	2069 0.243 0.135	− − −
Menshikova et al., 2005 [45] Females	mtDNA, Rc Succinate oxidase, U/mU CK ETC/mtDNA ratio	1591 0.124 0.086	1711 0.176 0.118	− − −
Menshikova et al., 2006 [54]	mtDNA, Rc NADH oxidase U/mU CK SS Cardiolipin ug/mU CK IMF1 Cardiolipin ug/mU CK IMF 2 Cardiolipin ug/mU CK Succinate oxidase U * mU CK^−1^	1264 0.51 n/a (12) n/a (18) n/a (60) 0.13	1895 1.0 n/a (20) n/a (24) n/a (90) 0.20	+ + + − + +
Menshikova et al., 2007 [55]	mtDNA, Rc Cardiolipin ug/mU CK NADH oxidase U/mU CK Ubiquinol oxidase U/mU CK Succinate dehydrogenase U/mU CK CS U/mU CK	1.901 46.9 0.15 0.54 0.19 3.06	2.169 73.7 0.29 0.79 0.26 3.91	− + + + + +
Majerczak et al., 2012 [60]	mtDNA, Rc COX I Cytochrome C	6685 n/a n/a	5977 n/a n/a	− − −
Egan et al., 2013 [59]	mtDNA/nDNA ratio, ab CS protein µmol min^−1^ µg ^−1^ COX IV protein, ab Cytochrome C protein, ab PGC-1a protein, ab PGC-1a, mRNA	n/a (2.7) 1.0 n/a (13) n/a (6) n/a (6) n/a (0.9)	n/a (2.6) 1.60 n/a (17) n/a (11) n/a (10) n/a (1.15)	− + + + + +
Shute et al., 2020 [58]	mtDNA, Rc PGC-1a, mRNA	1,00 0.048	1.08 0.059	− −
Fritzen et al., 2019 [57]	mtDNA, mtDNA/nDNA ratio CS, mU/mg protein Cardiolipin, ab Porin, a-tabulin, ab Complex I, mU/mg muscle Complex II, mU/mg muscle Complex III, mU/mg muscle Complex IV, mU/mg muscle	n/a (1200) 308 n/a (35) n/a (0.8) n/a (105) n/a (120) n/a (350) n/a (1100)	n/a (1600) 465 n/a (40) n/a (1.0) n/a (155) n/a (175) n/a (570) n/a (1800)	− + − − + + + +
Murakami et al., 2002 [56]	mtDNA/18S, ab CS, nmol/min/g tissue	35.6 12.1	44.0 17.4	+ +
Toledo et al., 2008 [51]	mtDNA, Rc Caridolipin µg/CK NADH oxidase U/mU CK mt size TEM µm^2^ mt density% TEM	2.049 n/a (70) n/a (0.18) n/a (0.070) n/a (4%)	2.185 n/a (95) n/a (0.3) n/a (0.065) n/a (6%)	− + + − +

All data are represented as means. Variability measures are excluded from the table due to substantial dissimilarity in variability measures. In third and fourth column, for the results presented as n/a, the subsequent value given in parenthesis is an expression that the value has been extracted from figures given in the articles. Thus, the numbers were not given in the text or table and may therefore be subject to small errors. + = change (*p* < 0.05); − = No change (*p* > 0.05). Abbreviations: mtDNA = Mitochondrial DNA, ETC = Electron transport chain, CK = Creatine kinase, COX IV = Cytochrome C oxidase, COX I = Cytochrome C oxidase subunit I, SS = Subsarcolemmal fractions, IMF1 + IMF2 = Intermyofibrillar fractions, CS = Citrate synthase, ab = Arbitrary unit, Rc = Copy number of mtDNA relative to nuclear genome.

## 5. Effects of Training on Biomarkers of Mitochondrial Function and Content (Table 4)

### 5.1. Citrate Synthase

Four out of nine studies measured citrate synthase, and all four found that moderate-to-high-intensity aerobic exercise resulted in an increase in citrate synthase levels [56,57,58,60]. 

### 5.2. PGC-1a

Two of the analysed studies measured the effect of moderate-to-high-intensity exercise on PGC-1*a* levels [58,59]. In the study by Egan et al., healthy subjects exercised for 60 min at an intensity of ~80% VO_2peak_ for 14 consecutive days [59]. Muscle biopsies were performed 16 h after cessation of the last exercise session. The authors found an impact on PGC-1*a* proteins and PGC-1*a* mRNA [59]. In another study, healthy subjects performed 60 min of exercise daily for 18 days at an intensity of 15 on the rated perceived exertion scale (RPE) [58]. This study performed muscle biopsies after 4 h of recovery from the last exercise bout and did not find any impact on PGC-1*a* mRNA level [58].

### 5.3. Cardiolipin 

Four studies measured cardiolipin [48,54,55,57]. Three of these studies revealed improvements in cardiolipin [48,54,55]. In the study by Toledo et al., there was an exercise-induced increase in cardiolipin that proportionally translated into an increase in mitochondrial density measured by TEM after 16–20 weeks of aerobic exercise, at an intensity of 60–70% of maximal heart rate [48].

### 5.4. Electron Transport Chain Complex Activities

Markers for complex activities were reported in seven out of nine studies [45,48,54,55,57,59,60]. Biomarkers for complex activities included NADH oxidase, succinate dehydrogenase, ubiquinol oxidase and cytochrome C. Five studies reported improvements in at least one of the biomarkers for complex activities in response to aerobic exercise [48,54,55,57,59]. Three studies measured NADH oxidase, and all three found an exercise-induced increase in NADH oxidase [48,54,55].

## 6. Effects of Training Intervention on Oxygen Uptake and Insulin Resistance (Table 5 and Table 6)

As presented in Table 5, eight of nine studies achieved improvements in oxygen uptake [45,51,54,55,56,57,58,59]. Insulin resistance was measured in four of nine studies [45,51,54,55]. Improvements were revealed in three out of these four studies [45,48,54] (Table 3). However, in one of these studies the improvement was only present in the female participants [45] (Table 3).

**Table 5:** Effects of aerobic exercise on oxygen uptake.

Study	Units	VO_2Max/Peak_ Pre	VO_2Max/Peak_ Post	Change
Menshikova et al., 2005 [45] Males	VO_2max_, mL ∗ kg FFM^−1^ ∗ min^−1^	39.0	48.7	+
Menshikova et al., 2005 [45] Females	VO_2max_, mL ∗ kg FFM^−1^ ∗ min^−1^	40.2	47.0	+
Menshikova et al., 2006 [54]	VO_2max_, L/min	1.64	1.88	+
Menshikova et al., 2007 [55]	VO_2max_, mL ∗ kg FFM^−1^ ∗ min^−1^	36.5	44.7	+
Majerczak et al., 2012 [60]	VO_2max_, mL ∗ min^−1^	3480	3536	−
Egan et al., 2013 [59]	VO_2peak_, L min^−1^	2.81	3.30	+
Shute et al., 2020 [58]	VO_2peak_, L min^−1^	3.34	3.72	+
Fritzen et al., 2019 [57]	VO_2peak_, mL ∗ min^−1^	1855	2475	+
Murakami et al., 2002 [56]	VO_2max_, mL/min/kg	40.7 *	46.6 *	+
Toledo et al., 2008 [51]	VO_2max_, mL ∗ min^−1^ ∗ kg LBM^−1^	47.6	52.4	+

+ = change (*p* < 0.05); − = No change (*p* > 0.05). * Data representing n = 29 who underwent mtDNA measures.

**Table 6:** Insulin resistance.

Study	Units	Preintervention	Postintervention	Change
Menshikova et al., 2005 [45] Males	Clamp, RQ	0.90	0.89	−
Menshikova et al., 2005 [45] Females	Clamp, RQ	0.89	0.93	+
Menshikova et al., 2006 [54]	HOMA:IR	3.05	2.32	+
Menshikova et al., 2007 [55]	Clamp, RQ	0,.89	0.92	−
Toledo et al., 2008 [51]	Hyperinsulinemic–euglycenic clamp	89.7 ± 6.6 µU/mL P:insulin	80.7 µU/mL P:insulin	+

All data are represented as means. Variability measures are excluded from the table due to substantial dissimilarity in variability measures. + = change (*p* < 0.05); − = No change (*p* > 0.05). Abbreviations: RQ = Insulin-stimulated respiratory quotient (RQ).

## 7. Discussion

This scoping review aimed to summarize the studies that have used mtDNA as a biomarker for mitochondrial adaptions and to address to what extent mtDNA resembles mitochondrial content in response to aerobic exercise in the healthy skeletal muscle. The findings of this review suggest that mitochondrial adaptions in response to moderate–high-intensity aerobic exercises occur without an increase in skeletal muscle mtDNA.

### 7.1. Response to Exercise: Maximal Oxygen Uptake

Maximal oxygen uptake (VO_2max_) is a marker for cardiorespiratory fitness, where O_2_ delivery is the limiting factor for VO_2max_ [63]. One effective way to improve oxidative capacity in the skeletal muscle is through aerobic exercise [64,65,66]. Aerobic exercise necessitates increased ATP demand in the skeletal muscle, which requires an increase in mitochondrial enzyme activity to meet the increased ATP demand [35,41,64]. Since mitochondria appear to have a central role in aging, cell pathology and metabolic disorders (3.4), exercise has been used to uncover adaptations of the mitochondria in response to higher oxidative demand [67,68]. This review investigated if biomarkers of mitochondrial content improve in response to aerobic exercise without changes in mtDNA levels. Eight out of nine studies included in this review found improvements in oxygen uptake postintervention [45,51,54,55,56,57,58,59]. Six of these studies additionally found increases in biomarkers of mitochondrial adaptions [51,54,55,56,57,59]. One of the included studies that achieved improvements in VO_2max_ and simultaneously did not reveal any improvements in mitochondrial biomarkers used mtDNA and PGC-1*a* mRNA as markers for mitochondrial adaptions [58]. This study obtained muscle biopsies four hours after the last training session [58]. It has been documented that PGC-1*a* mRNA levels are most pronounced if measured two hours after cessation of the exercise session and that PGC-1*a* mRNA levels markedly decrease if muscle biopsies are performed even six hours after cessation of the exercise session [69]. Thus, the absence of improvements in markers for mitochondrial adaptions in this particular study could be explained by the choice of biomarkers for mitochondrial adaptions.

Only one study did not show any improvements in VO_2max_ in response to the exercise intervention [60]. Correspondingly, this study did not demonstrate any change in mtDNA content or ETC activity after five weeks of aerobic exercise [60]. The absence of improvements in mitochondrial biomarkers for this study is most likely attributed to the missing improvement in aerobic capacity.

### 7.2. Response to Exercise: Citrate Synthase

Citrate synthase is an enzyme located in the mitochondria and is essential in mitochondrial metabolism [23]. An association between aerobic capacity and citrate synthase has been demonstrated in observational studies [70]. It has been suggested that training volume is one of the main determinants of exercise-induced improvements in citrate synthase and that training intensity, to a greater extent, relates to improvements in mitochondrial respiration more than in citrate synthase [11,25]. However, studies have suggested that the intensity of training affects citrate synthase activity [25,71], where the number of training bouts (frequency) alone does not seem to have an impact in the level of citrate synthase [72,73].

Four of the nine included studies reported citrate synthase activity, and all four studies found an increase in citrate synthase after aerobic exercise [55,56,57,59]. The increase was similar in all studies, despite a large difference in the number of training bouts per week, i.e., 3.5 days to 7 days a week [55,56,57,59]. Therefore, although statistically, sub-analysis could not be performed, the findings imply that training frequency itself does not have an impact on citrate synthase level. Due to the similar training intensity in all studies, a potential impact of intensity on citrate synthase level cannot be addressed. It has been demonstrated that citrate synthase correlates with mitochondrial content using TEM and is therefore valid as a biomarker for changes in mitochondrial content [74]. The findings from this study support this assumption, given that all four studies that measured citrate synthase found improvements. Only one of these studies additionally found an exercise-induced increase in mtDNA [56]. Taken together, these studies indicate that citrate synthase can increase without a corresponding change in mtDNA.

### 7.3. Response to Exercise: Mitochondrial Volume (Porin and Cardiolipin)

Porin is a protein present in the outer mitochondrial membrane, whereas cardiolipin is a phospholipid located in the inner membrane of the mitochondria, and both have been used as a quantitative marker for mitochondrial membrane surface area, also expressed as mitochondrial volume [13,14,15]. The mitochondrial inner membrane, also known as the mitochondrial cristae, was previously thought not to be very plastic; however, one cross-sectional study compared sedentary subjects with trained subjects and identified a difference in the mitochondrial cristae in the trained subjects compared with the sedentary subjects [75]. This finding indicated, that the mitochondrial inner membrane is not constant, but exhibits plasticity in response to long-term endurance training and could be a mitochondrial adaption to meet the increased exercise-induced ATP demand [75]. The adaptions of cardiolipin in response to exercise have been investigated in interventional studies that have tended towards showing that exercise leads to an increase in cardiolipin levels [48,76,77]. The increase in mitochondrial inner membrane, e.g., cardiolipin, has been hypothesized to provide infrastructure for more ETC activity, denoting increased surface area increases the enzymatic activity of the ETC and is thought to be a central role in mitochondrial biogenesis [55]. 

Cardiolipin has been demonstrated to be the biomarker that has the strongest correlation with mitochondrial content, closely followed by citrate synthase [74]. There has not been a procedure to measure cardiolipin in small muscle biopsies (<100 mg) [55], whereas citrate synthase can easily be measured in small skeletal muscle samples (10 mg of tissue) and can be measured in the same solution as used for mitochondrial respiration [78]. Cardiolipin was measured in four out of nine studies included in this review [51,54,55,57]. Improvements with exercise were identified in three of four studies [51,54,55]. One of the four studies additionally measured porin, and did not reveal an increase in either cardiolipin or porin [57]. In one study, where an increase was found in cardiolipin, mtDNA copy number additionally increased [54]. Another study found increasing levels of cardiolipin and a proportional increase in mitochondrial density measured by TEM [51]. This study did not simultaneously find any improvements in mtDNA [51]. Since TEM is thought to be the gold standard for assessing mitochondrial adaptions, this indicates that mitochondrial adaptions can occur independent of mtDNA levels. These findings indicate that the mitochondrial inner membrane adapts to aerobic exercise and that the adaption occurs independently of mtDNA levels.

### 7.4. Response to Exercise: Mitochondrial Complex Activities

Mitochondrial complex activities (ETC) play an essential role in oxidizing substrates, such as glucose and fatty acids, to ATP. The limiting factor for ETC assembly in the mitochondria appears to depend on the availability of transcripts for mitochondrial proteins encoded by nuclear DNA, rather than mtDNA transcripts [79,80]. Biomarkers of ETC were measured in six out of nine studies [45,51,54,55,57,60]. Four of these studies found improvements in ETC after aerobic exercise [51,54,55,57], and three of these studies did not find changes in mtDNA [51,55,57]. Only one study revealed an increase in mtDNA levels simultaneously with increased ETC activity [54]. In all, these findings indicate that ETC levels can increase independently of mtDNA.

### 7.5. Response to Exercise: mtDNA

A previous study identified a correlation between citrate synthase and mtDNA levels in skeletal muscle biopsies [44]. Subsequently, mtDNA has been widely applied as a biomarker for improved mitochondrial content in response to exercise [45,46,47,48]. In contrast, other studies have indicated that mitochondrial biomarkers, such as citrate synthase, ETC and cardiolipin can improve without a change in mtDNA levels [49,50,51]. Various factors have been recognized to have an impact on mtDNA levels: in obese subjects, mtDNA levels was found to be reduced by 25% when compared with lean subjects [81]; individual differences in mtDNA copy number per cell can vary between 4–6 copies per cell [74,82]; and assay methodology to assess mtDNA copy number [83,84]. Only two of the nine included studies revealed an increase in mtDNA levels in response to aerobic exercise [54,56], whereas improvements in biomarkers for mitochondrial adaptions were found in six of nine included studies [51,54,55,56,57,59]. These findings indicate that biomarkers for mitochondrial content can increase simultaneously with unchanged mtDNA levels as a response to aerobic exercise. The interference of deconditioning, correct assay methodology, overweight and insulin resistance in mitochondrial content and mtDNA levels will be discussed below.

### 7.6. Response to Deconditioning: Citrate Synthase, ETC and mtDNA

Mitochondria are very plastic organelles that adapt rapidly to exercise and deconditioning [25,85]. Thus, a way to examine the effect of training on mitochondrial content is to examine the impact of deconditioning in mitochondrial biomarkers. Only one study has investigated citrate synthase, mtDNA and complex activities in response to four weeks of deconditioning that followed six weeks of vigorous knee extensor exercise [57]. Despite a substantial improvement in citrate synthase and complex activities after six weeks of knee extensor exercise, and a substantial decrease during the deconditioning phase, mtDNA did not change with either of the interventions [57]. This finding indicates that changes in citrate synthase and complex activities are very adaptable to aerobic exercise, but also that the improvements depend on the exercise activity being maintained and that these mitochondrial adaptions can occur without any changes in mtDNA.

### 7.7. Assay Methodology

mtDNA copy number can be measured using different techniques, i.e., Southern blotting and real-time PCR (qPCR). The qPCR technique is the overall gold standard for assessing mtDNA copy number [86]. mtDNA copy number quantification should be addressed as mtDNA copies per cell or per diploid genome, where copy number ideally should be corrected for the copy number of nuclear reference gene [83,87]. Several nuclear genes can be used as reference genes, where examples of single copy number nuclear genes are: RPP30, beta-globin, 28S rRNA and 18S rRNA [83]. Copies per cell can vary depending on which gene that is used as the reference gene, and challenge comparisons between studies where there is no consistency in the nuclear reference gene [83]. In the included studies, only two of the nine studies found an increase in mtDNA as a response to vigorous aerobic exercise [54,56]. One of these studies used 18S rRNA as a nuclear reference gene [56]. Not many of the included studies reported reference nuclear genes, and specific 18S rRNA was only reported as reference gene in this particular study. A potential reason for finding an increase in mtDNA in response to aerobic exercise for this study could be due to the choice of this specific nuclear gene (18S rRNA), since RNA is not as constant as other nuclear genes.

### 7.8. Mitochondrial Content in Skeletal Muscle: Obesity 

Studies have implied that mitochondrial size, mtDNA and citrate synthase activity is reduced in the skeletal muscle of obese subjects [81,88,89]. Theoretically, a low mtDNA level at baseline could potentially influence the ability of mtDNA transcripts to respond to alterations in mitochondrial content, as with aerobic exercise. This could indicate that obese subjects may not be as capable of inducing the same amount of mtDNA transcripts as lean subjects, whereas the postinterventional mtDNA transcripts might not be as notable as in lean subjects due to fewer transcripts. In this review, three of the included studies comprehended obese subjects (BMI > 30) [45,51,55]. These studies performed mtDNA measures before and after 12–20 weeks of aerobic exercise and did not find any increase in mtDNA postintervention [45,51,55]. It could be hypothesized that the lack of change in mtDNA was caused by an impaired ability of mtDNA transcripts in response to aerobic exercise. However, two of these studies revealed an increase in other markers of mitochondrial content and volume, i.e., ETC, cardiolipin, citrate synthase and mitochondrial density % TEM, despite unchanged mtDNA levels postintervention [51,55]. None of these studies used a control group of lean subjects, which makes it difficult to determine if the lack of increase in mtDNA copy number postintervention is caused by impaired ability for mtDNA transcripts due to obesity. However, it is possible that the absence of a change in mtDNA levels can be attributed to insufficient aerobic exercise volume, since obese subjects have increased body mass, that prevents them from exercising as efficiently as lean subjects. The increases in ETC, cardiolipin, citrate synthase and mitochondrial density % TEM indicate that improved mitochondrial content and volume can be achieved in overweight subjects despite unchanged mtDNA levels. Three of the studies included in this review, evaluated normal weight subjects with BMIs ranging from 22–23.6 kg/m^2^ [57,59,60]. These subjects performed moderate–high-intensity aerobic exercise for 5–6 weeks [57,59,60]. None of these studies found an increase in mtDNA, whereas two studies found an increase in ETC, citrate synthase, PGC-1*a* mRNA and PGC-1*a* proteins [57,59]. These findings indicate that improvements in mitochondrial content and volume can occur despite unchanged mtDNA and differences in BMI.

### 7.9. Mitochondrial Content in Skeletal Muscle: Insulin Resistance

Studies have suggested that there might be an association between insulin resistance and impaired mitochondrial function. This notion is based on findings that OXPHOS can be up to 40% reduced in insulin-resistant subjects, when compared with healthy subjects [90,91,92]. An increase in mtDNA levels was only present in two of the nine included studies [54,56]. In one of these studies, insulin measurements were conducted pre- and postintervention [54]. These authors found improvements in insulin resistance assessed by HOMA index, which improved from 3.0 to 2.3 [54]. It could be hypothesized, that the increase in mtDNA in response to aerobic exercise in this study was induced by an improvement in insulin resistance. This study, proportionally found improvements in ETC activity [54]. However, the use of the HOMA index as a measure of insulin resistance in interventional studies is not accurate [93]. Additionally, two of the included studies also measured insulin resistance assessed by clamp and found improvements in insulin resistance after 16–20 weeks of aerobic exercise [45,51]. These two studies did not reveal any increase in mtDNA copy number [45,51], whereas one of the studies did find improvements in ETC, mitochondrial density % TEM and cardiolipin [51]. These findings suggest that improvements in ETC and cardiolipin can occur with unchanged mtDNA levels and that insulin resistance does not necessarily influence mtDNA copy number. This should be interpreted with caution, due to the differences in assessing insulin resistance, small sample sizes and the missing control groups. The link between insulin resistance and OXPHOS has been questioned, where the dominant player is suggested to be improved physical activity [94].

## 8. Conclusions

The included literature in this review indicates that mitochondrial volume, expressed as cardiolipin, can increase without corresponding changes in mtDNA levels and that improvements in mitochondrial function, expressed as citrate synthase and complex activities, can occur without an increase in mtDNA levels.

## 9. Limitations

This scoping review has several limitations. The included studies were primarily based on non-controlled trials. The absence of control groups makes it challenging to determine if mitochondrial function and content changes were by coincidence. Moreover, small sample sizes dominated the studies; five out of nine studies had fewer than ten participants. The small sample sizes increase the risk that the conclusions and findings of the present study are found by coincidence. Only one included study performed a power calculation [57]. The lack of power calculations and small sample sizes reduces the likelihood that the findings reflect the actual effect [95]. A further limitation is that the studies applied different nuclear reference genes, which can affect individual differences in mtDNA estimations and complicate comparisons between the studies.

## Data Availability

Not applicable.

## Appendix A. Search Strategy

Databases: PubMed and Embase 12/12/2022.

A combination was used of: MeSH terms/Mapterms, Title/abstract/keywords

### Appendix A.1. PubMed

DNA, Mitochondrial [*MESH*]
“Mitochondrial DNA” [*Title*/*Abstract*]
“Mitochondrial DNA content” [*Title*/*Abstract*]
“mtDNA” [*Title*/*Abstract*]
“Mitochondrial DNA copy number” [*Title*/*Abstract*]
“mtDNA copy number” [*Title*/*Abstract*]
#1 OR #2 OR #3 OR #4 OR #5 OR #6 [*Hits*: 60,614]
Aerobic Exercise [*MESH*]
“High-Intensity Interval Training” [*MESH*]
“Endurance Training” [*MESH*]
Exercise test “ [*MESH*]
Exercise [*Title*/*Abstract*]
Training [*Title*/*Abstract*]
#8 OR #9 OR #10 OR #11 OR #12 OR #13 [*Hits*: 905,153]
#7 AND #14 [*Hits*: 820]

### Appendix A.2. Embase

Mitochondrial DNA/[*Map term*]
mtDNA [*Keyword*]
Mitochondrial DNA copy number [*Keyword*]
mtDNA copy number [*Keyword*]
Mitochondrial DNA [*Keyword*]
#1 OR #2 OR #3 OR #4 OR #5 [*Hits*: 71,338]
Aerobic Exercise/[*Map term*]
Exercise/[*Map term*]
High intensity interval training/[*Map term*]
#7 OR #8 OR #9 [*Hits*: 342822]
#6 AND #10 [*Hits*: *765*]
